# ZEB1 induces ER-*α* promoter hypermethylation and confers antiestrogen resistance in breast cancer

**DOI:** 10.1038/cddis.2017.154

**Published:** 2017-04-06

**Authors:** Jianbo Zhang, Chen Zhou, Huimin Jiang, Lin Liang, Wen Shi, Quansheng Zhang, Peiqing Sun, Rong Xiang, Yue Wang, Shuang Yang

**Affiliations:** 1 Department of Gastrointestinal Surgery, Second Affiliated Hospital of Chongqing Medical University, Chongqing 400010, China; 2 Department of Medical Genetics, Tianjin Key Laboratory of Tumor Microenvironment and Neurovascular Regulation, Medical College of Nankai University, Tianjin 300071, China; 3 2011 Project Collaborative Innovation Center for Biotherapy of Ministry of Education, Medical College of Nankai University, Tianjin 300071, China; 4 Tianjin Key Laboratory of Organ Transplantation, Tianjin First Center Hospital, Tianjin 300192, China; 5 Department of Cancer Biology, Wake Forest University School of Medicine, Winston-Salem, NC 27157, USA

## Abstract

Antiestrogen resistance is a major obstacle to endocrine therapy for breast cancers. Although reduced estrogen receptor-*α* (ER-*α*) expression is a known contributing factor to antiestrogen resistance, the mechanisms of ER-*α* downregulation in antiestrogen resistance are not fully understood. Here, we report that ectopic zinc-finger E-box binding homeobox 1 (ZEB1) is associated with ER-*α* deficiency in breast cancer cells and thus confers antiestrogen resistance. Mechanistically, ZEB1 represses ER-*α* transcription by forming a ZEB1/DNA methyltransferase (DNMT)3B/histone deacetylase (HDAC)1 complex on the ER-*α* promoter, leading to DNA hypermethylation and the silencing of ER-*α*. Thus, ectopic ZEB1 downregulates ER-*α* expression and subsequently attenuates cell growth inhibition by antiestrogens, such as tamoxifen and fulvestrant. Notably, the depletion of ZEB1 by RNA interference causes ER-*α* promoter demethylation, restores ER-*α* expression, and increases the responsiveness of breast cancer cells to antiestrogen treatment. By studying specimens from a large cohort of subjects with breast cancer, we found a strong inverse correlation between ZEB1 and ER-*α* protein expression. Moreover, breast tumors that highly express ZEB1 exhibit ER-*α* promoter hypermethylation. Using a nude mouse xenograft model, we further confirmed that the downregulation of ZEB1 expression restores the responsiveness of breast cancer cells to antiestrogen therapy *in vivo*. Therefore, our findings suggest that ZEB1 is a crucial determinant of resistance to antiestrogen therapies in breast cancer.

Estrogen receptor-*α* (ER-*α*) is a key transcriptional regulator that plays critical roles in normal breast development and breast tumorigenesis.^1, 2, 3, 4^ Approximately 70% of breast cancers are ER-*α* positive and are treated with targeted antiestrogen therapy by using effective ER blocking agents,^5, 6, 7^ such as tamoxifen and fulvestrant. Thus, ER-*α* expression is an important prognostic marker that is predictive for tumor response to antiestrogen treatment. However, intrinsic or acquired resistance to antiestrogen therapy presents a major challenge.^8, 9, 10, 11^ Antiestrogen resistance is believed to be caused primarily by alterations in the expression and function of ER-*α*.^12, 13^ Therefore, an improved understanding of the molecular mechanisms that control ER-*α* activity may reveal new molecular targets that could be exploited to more effectively treat and eradicate breast cancers.

While deletions, insertions, rearrangements, or polymorphisms of the *ER-α* gene are uncommon and are not generally associated with loss of ER-*α*,^14, 15^ there is increasing evidence that ER-*α* deficiency is a result of CpG island hypermethylation within the ER-*α* promoter.^16, 17, 18, 19^ An abnormal methylation pattern can account for the transcriptional inactivation of the *ER-α* gene and consequent antiestrogen resistance in breast cancer cell lines and tumors. Other epigenetic events, such as histone deacetylation, are also involved in the complex mechanisms that regulate the transcription of the ER-*α* promoter.^20, 21^ Notably, DNA methyltransferase (DNMT) and histone deacetylase (HDAC) inhibitors, which are candidates for new cancer therapeutics, can synergistically reactivate ER-*α* expression in ER-*α*-negative breast cancer cells and restore responsiveness to antiestrogen therapy.^22^

Zinc-finger E-box binding homeobox 1 (ZEB1) is a crucial member of the zinc-finger-homeodomain transcription factor family involved in the regulation of cell fate determination^23, 24^ and in the differentiation of several lineages, such as in myogenesis^25^ and osteogenesis.^26^ Beyond its physiological roles, ZEB1 is overexpressed in breast cancers,^27, 28^ regulates cell adherence and polarity,^29, 30^ modulates chemoresistance and radioresistance,^24, 31^ and promotes the generation of breast cancer stem cells.^32, 33^ Notably, a growing body of evidence has implied a potential role of ZEB1 in epigenetic regulation during tumorigenesis.^34, 35, 36^ For example, ZEB1 interacts with HDAC1 and HDAC2 at the E-cadherin promoter in either an miR-200-dependent^35^ or an miR-200-independent manner,^34, 36^ resulting in the transcriptional silencing of E-cadherin and cancer progression. However, the cellular and molecular mechanisms by which ZEB1 regulates dynamic epigenetic modification remain incompletely understood.

In this study, we demonstrate a mechanism for ZEB1/ER-*α*-mediated antiestrogen resistance in breast cancer by which ER-*α* promoter methylation and histone deacetylation are established. ZEB1 represses ER-*α* expression by forming a ZEB1/DNMT3B/HDAC1 complex on its promoter. Notably, the downregulation of ZEB1 restores ER-*α* activity and thus increases the sensitivity of breast cancer cells to antiestrogen treatment *in vitro* and *in vivo*. We therefore suggest that ZEB1 or pathways downstream of ZEB1 may be viable therapeutic targets. The inhibition of ZEB1 to restore ER-*α* expression, in combination with methylation inhibitors and/or HDAC inhibitors, will represent a new strategy for overcoming antiestrogen resistance in breast cancer.

## Results

### Ectopic expression of ZEB1 results in the promoter hypermethylation and silencing of ER-*α* in breast cancer cells

**Figure 1:**
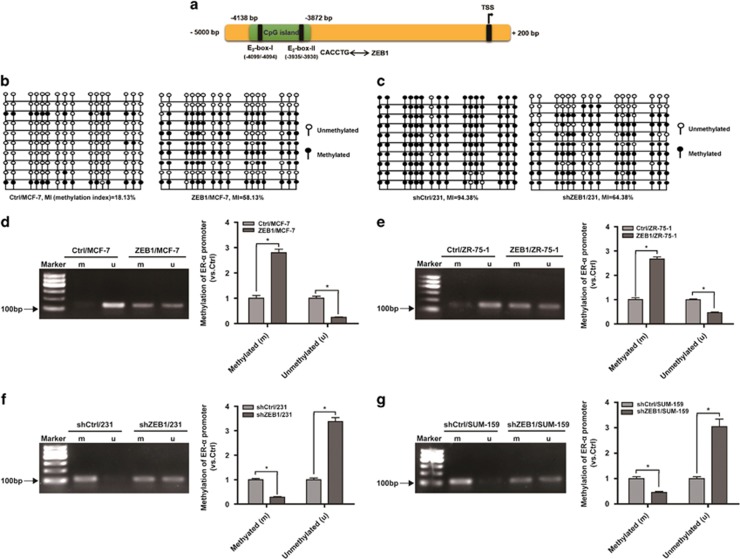
Ectopic ZEB1 increases DNA methylation of the ER-*α* promoter. (**a**) An upstream CpG-rich region was located at position −4138/−3872 of the ER-*α* promoter, and two canonical E_2_-box elements for ZEB1 binding were identified within. (**b** and **c**) Percentage of DNA methylation of the ER-*α* promoter was determined by bisulfite sequencing PCR (BSP) in ZEB1/MCF-7 *versus* Ctrl/MCF-7 cells (**b**) and in shZEB1/231 *versus* shCtrl/231 cells (**c**). (**d** and **e**) Basal methylation levels of the ER-*α* promoter were determined by methylation-specific PCR (MSP) in ZEB1/MCF-7 *versus* Ctrl/MCF-7 cells (**d**) and in ZEB1/ZR-75-1 *versus* Ctrl/ZR-75-1 cells (**e**). *P<0.05 *versus* the respective control in Student's *t*-test. (**f** and **g**) Basal methylation levels of the ER-*α* promoter were determined by MSP in shZEB1/231 *versus* shCtrl/231 cells (**f**) and in shZEB1/SUM-159 *versus* shCtrl/SUM-159 cells (**g**). *P<0.05 *versus* the respective control in Student's *t*-test

To determine whether ZEB1-regulated ER-*α* expression in breast cancer is correlated with DNA methylation, we performed a search using the CpG island prediction database MethPrime and identified an upstream CpG-rich region at position −4138/−3872 of the ER-*α* promoter (Figure 1a). Two canonical E_2_-box elements (CACCTG) to which ZEB1 can potentially bind were found within this region.^29, 37^ ZEB1 gain-of-function experiments in MCF-7 cells and loss-of-function experiments in MDA-MB-231 cells were then performed using lentiviruses (Supplementary Figures S1a and b). The methylation status of 16 CpG residues in the 267-bp region of the ER-*α* promoter was interrogated using bisulfite sequencing PCR. Relative to Ctrl/MCF-7, an increase in DNA methylation was detected in ZEB1/MCF-7 cells (Figure 1b). In contrast, ZEB1 knockdown in MDA-MB-231 showed an opposite effect of decreased DNA methylation (Figure 1c). Methylation-specific PCR analysis further revealed that ZEB1 overexpression in two luminal (MCF-7 and ZR-75-1) breast cancer cell types enhanced DNA methylation of the ER-*α* promoter (Figures 1d and e; Supplementary Figure S1c), whereas ZEB1 knockdown in two basal (MDA-MB-231 and SUM-159) breast cancer cell types (Figures 1f and g; Supplementary Figure S1d) reduced this methylation.

**Figure 2:**
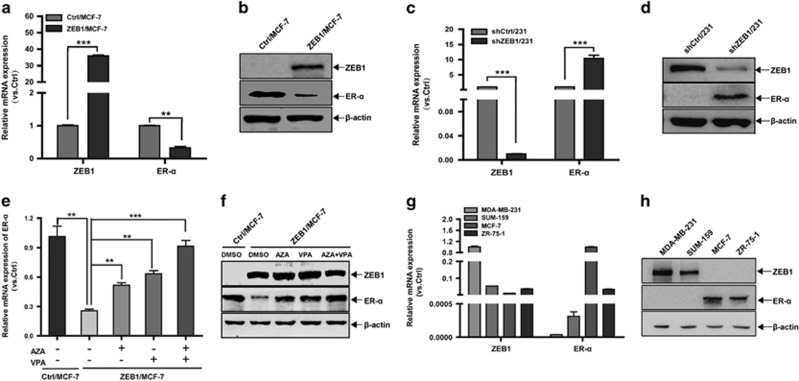
Ectopic ZEB1 downregulates ER-*α* expression. (**a** and **b**) The expression of ZEB1 and ER-*α* in ZEB1/MCF-7 and Ctrl/MCF-7 cells was assessed by quantitative PCR (**a**) and immunoblotting (**b**) and normalized to the levels of *β*-actin. **P<0.01, ***P<0.001 *versus* the respective control in Student's *t*-test. (**c** and **d**) The expression of ZEB1 and ER-*α* in shZEB1/231 and shCtrl/231 cells was assessed by quantitative PCR (**c**) and immunoblotting (**d**) and normalized to the levels of *β*-actin. ***P<0.001 *versus* the respective control in Student's *t*-test. (**e** and **f**) ZEB1/MCF-7 and Ctrl/MCF-7 cells were treated with AZA (1.5 *μ*M) and/or VPA (1.5 mM) for 72 h. The expression of ZEB1 and ER-*α* was assessed by quantitative PCR (**e**) and immunoblotting (**f**) and normalized to the levels of *β*-actin. **P<0.01, ***P<0.001 *versus* the respective control in Student's *t*-test. (**g** and **h**) The expression levels of ZEB1 and ER-*α* in two basal (MDA-MB-231 and SUM-159) and two luminal (MCF-7 and ZR-75-1) breast cancer cell lines were assessed by quantitative PCR (**g**) and immunoblotting (**h**) and normalized to the levels of *β*-actin

Subsequently, we sought to assess ZEB1-regulated ER-*α* expression at the messenger RNA and protein levels in ZEB1/MCF-7 (Figures 2a and b) and shZEB1/231 cells (Figures 2c and d) by qPCR and immunoblotting, and we demonstrated the downregulation of ER-*α* expression by ZEB1. Mechanistically, gene regulation via promoter methylation is accompanied, in some cases, by an increase in the activities of DNMT and HDAC.^20, 21, 22^ Thus, ZEB1/MCF-7 cells were treated with the demethylating agent 5-aza-2′-deoxycytidine (AZA), the HDAC inhibitor valproic acid (VPA), or a combination of the two. The results indicated that treatment with either AZA or VPA abolished the ZEB1-mediated downregulation of ER-*α* at the messenger RNA and protein levels (Figures 2e and f). The combination of AZA and VPA rescued ER-*α* to a greater degree than either agent alone.

To confirm this finding, breast cancer cell lines were evaluated for ZEB1 and ER-*α* expression. The results revealed an inverse correlation between ZEB1 and ER-*α* at both messenger RNA and protein levels within the cell lines tested (Figures 2g and h). Collectively, the above observations indicate a potential role for ZEB1 in the regulation of ER-*α* promoter hypermethylation, thus altering ER-*α* expression in breast cancer cells.

### ZEB1 represses ER-*α* transcription by recruiting DNMT3B and HDAC1 to the ER-*α* promoter

**Figure 3:**
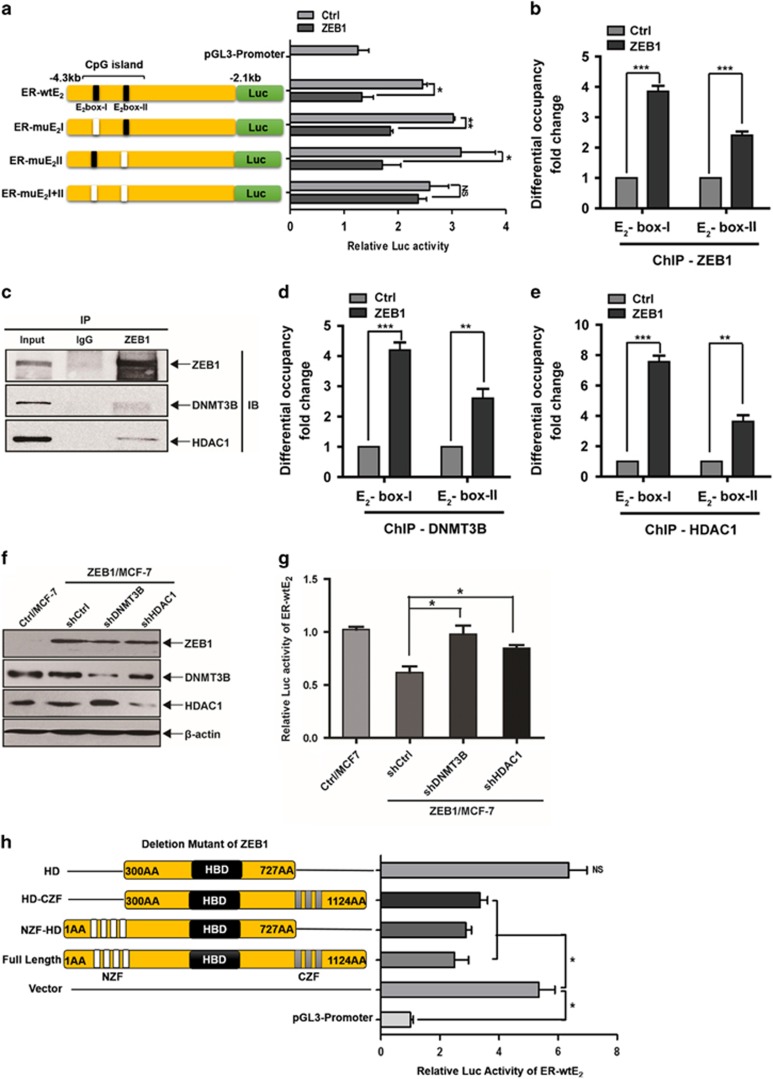
ZEB1 represses ER-*α* transcription via interaction with DNMT3B and HDAC1. (**a**) MCF-7 cells were co-transfected with the ZEB1 expression plasmid and different wild-type or mutant ER-*α* promoter luciferase reporter constructs. Extract luciferase activities were determined 36 h after transfection using a Betascope analyzer. Luciferase values were normalized to Renilla activities. *P<0.05, **P<0.01 *versus* the respective control in Student's *t*-test. (**b**) The overexpression of ZEB1 significantly enhanced its recruitment to the endogenous ER-*α* promoter, as confirmed by a quantitative ChIP assay using E_2_-box I- and E_2_-box II-specific primers. ***P<0.001 *versus* the respective control in Student's *t*-test. (**c**) The interactions among ZEB1, DNMT3B, and HDAC1 protein were analyzed by co-immunoprecipitation in ZEB1/MCF-7 cells. (**d** and **e**) ZEB1 overexpression significantly enhanced the recruitment of DNMT3B (**d**) and HDAC1 (**e**) to the endogenous ER-*α* promoter as confirmed by a quantitative ChIP assay using E_2_-box I- and E_2_-box II-specific primers. **P<0.01, ***P<0.001 *versus* the respective control in Student's *t*-test. (**f** and **g**) Specific shRNA targeting DNMT3B or HDAC1 was introduced into ZEB1/MCF-7 cells, which was followed by transfection with the ER-wtE_2_ reporter. (**f**) The expression of ZEB1, DNMT3B and HDAC1 was assessed by immunoblotting and normalized to the levels of *β*-actin. (**g**) Extract luciferase activities were determined 36 h after transfection using a Betascope analyzer. Luciferase values were normalized to Renilla activities. *P<0.05 *versus* the respective control in Student's *t*-test. (**h**) MCF-7 cells were co-transfected with the ER-wtE_2_ reporter and full-length or different ZEB1 deletion mutants. Extract luciferase activities were determined 36 h after transfection using a Betascope analyzer. Luciferase values were normalized to Renilla activities. *P<0.05 *versus* the respective control in Student's *t*-test

Next, we performed promoter-reporter assays to elucidate the molecular mechanism by which ZEB1 regulates ER-*α* transcription. As shown in Figure 3a, the wild-type -4184/-1951 promoter, ER-wtE_2_, has two canonical E_2_-box elements (CACCTG) at positions −4099/−4094 and −3935/−3930. The results of the luciferase assay indicated that ZEB1 overexpression decreased the promoter activity of the ER-wtE_2_ reporter by ~46% relative to the control without ZEB1 transfection in MCF-7 cells (Figure 3a). The E_2_-box elements were then manipulated by site-directed mutagenesis, individually or in combination. The luciferase assays showed that the mutation of either E_2_-box did not affect the ZEB1-mediated repression of ER-*α* promoter activity. However, simultaneous mutations within both E_2_-box elements completely eliminated the transcriptional repression of the ER-*α* promoter by ZEB1. In addition, the chromatin immunoprecipitation (ChIP) assays revealed that ZEB1 could bind to the ER-*α* promoter during basal conditions in an E_2_-box-dependent manner (Supplementary Figure S2). ZEB1 overexpression increased its binding to the endogenous ER-*α* promoter (Figure 3b). Moreover, significant binding was observed in the area of E_2_-box I. These observations indicated that ZEB1 binds directly or as part of a complex to the endogenous ER-*α* promoter, resulting in the transcriptional repression of ER-*α*.

To further elucidate the mechanism by which ZEB1 represses ER-*α* promoter activity, we investigated the recruitment of DNMT3B and HDAC1.^38^ The results showed that DNMT3B and HDAC1 each co-immunoprecipitated with ZEB1 in ZEB1/MCF-7 cells (Figure 3c). The ChIP experiments further demonstrated that DNMT3B and HDAC1 were recruited to both E_2_-box elements of the ER-*α* promoter, whereas maximum ChIP binding was seen in the area of E_2_-box I (Supplementary Figure S2; Figures 3d and e). Next, DNMT3B and HDAC1 expression levels were decreased by corresponding specific shRNAs (Figure 3f), and the ER-wtE_2_ reporter was transfected. Importantly, luciferase assays showed that the knockdown of DNMT3B or HDAC1 significantly attenuated ZEB1 repression of the ER-*α* promoter (Figure 3g). These observations point to an important role for the E_2_-box elements, especially E_2_-box I, in the regulation of ER-*α* by ZEB1 via interaction with DNMT3B and HDAC1.

Furthermore, we used ER-wtE_2_ and ZEB1 zinc-finger deletion mutants to determine the functional domains of ZEB1 involved in binding to the ER-*α* promoter. As observed in Figure 3h, the deletion of either zinc-finger domain did not affect the ZEB1-mediated repression of ER-*α* promoter activity, whereas simultaneous deletions within both zinc-finger domains completely abolished the transcriptional repression of the ER-*α* promoter by ZEB1. This finding indicated that ZEB1 binding to the ER-*α* promoter is abrogated when the functional zinc-finger domains are excised.

### ZEB1 is correlated with ER-*α* expression and promoter hypermethylation in breast cancer patients

**Figure 4:**
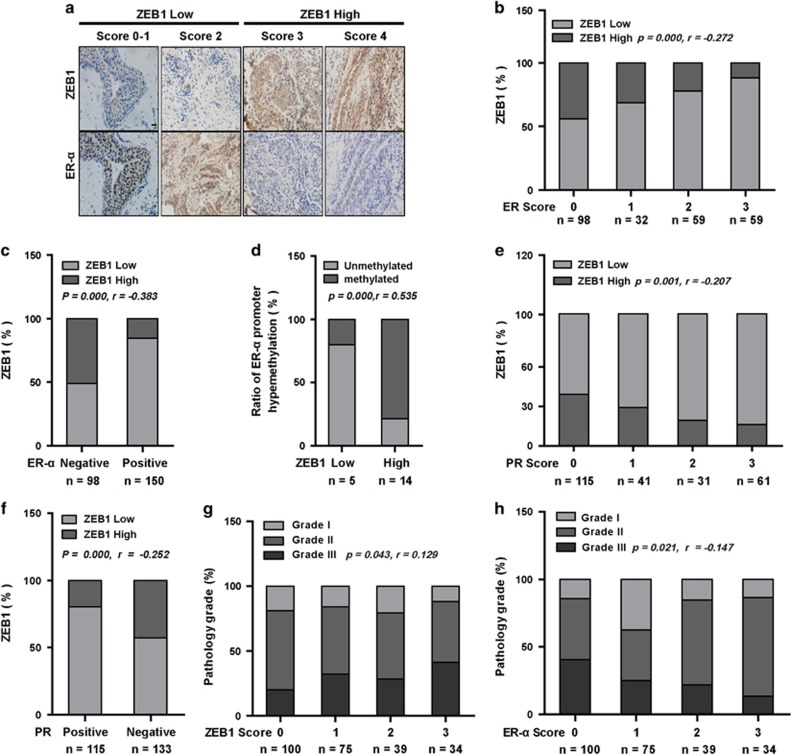
ZEB1 is correlated with ER-*α* expression and ER-*α* DNA promoter hypermethylation in human breast tumors. (**a**) Representative images of immunohistochemical staining of ZEB1 and ER-*α* in two serial sections of the same tumor from four cases are shown. Scale bars, 50 *μ*m. (**b** and **c**) The expression score (**b**, *P*=0.000, *r*=−0.272) and positive percentage analysis (**c**, *P*=0.000, *r*=-0.383) for ER-*α* indicate a negative correlation with ZEB1 expression in breast tumors from 248 subjects. Data were analyzed by Spearman's rank correction test. (**d**) DNA hypermethylation of the ER-*α* promoter is positively correlated with ZEB1 expression in 19 randomly selected samples. Data were analyzed by Spearman's rank correction test; *P*=0.000, *r*=0.535. (**e** and **f**) The expression score (**e**, *P*=0.001, *r*=−0.207) and positive percentage analysis (**f**, *P*=0.000, *r*=-0.252) for PR indicate a negative correlation with ZEB1 expression in breast tumors. Data were analyzed by Spearman's rank correction test. (**g**) The expression of ZEB1 is positively correlated with the histological grades of breast tumors. Data were analyzed by Spearman's rank correction test; *P*=0.043, *r*=0.129. (**h**) The expression of ER-*α* is negatively correlated with the histological grades of breast tumors. Data were analyzed by Spearman's rank correction test; *P*=0.021, *r*=−0.147

To better understand the correlation between ZEB1 and ER-*α*, we divided 248 cases of human breast carcinoma into two groups based on ZEB1 expression scores (Figure 4a). ER-*α* expression in each group was represented by its expression score or the percentage of positive cases. Our results demonstrated that ER-*α* expression was lower in tumors with high ZEB1 expression compared to tumors with lower ZEB1 expression (Figure 4b). Similarly, the positive percentage analysis for ER-*α* demonstrated a negative correlation with ZEB1 expression (Figure 4c). Importantly, ZEB1 expression was positively correlated with DNA hypermethylation of the ER-*α* promoter in 19 randomly selected samples (Figure 4d). We also examined the correlation between ZEB1 and PR, which is a downstream target of activated ER-*α*,^2, 3^ and obtained similar results (Figures 4e and f).

To further assess the clinical relevance of ZEB1 and ER-*α* downregulation, we investigated the relationship between ZEB1 and ER-*α* in different grades of tumors. The results showed that ZEB1 expression was relatively lower (Figure 4g) and that ER-*α* expression was higher (Figure 4h) in low-grade tumors compared to high-grade tumors, which is consistent with a previous report showing that low ER-*α* expression is often associated with high-grade breast cancer tumors and clinical resistance to hormone therapy.^39^

### Ectopic expression of ZEB1 confers antiestrogen resistance in breast cancer cells

**Figure 5:**
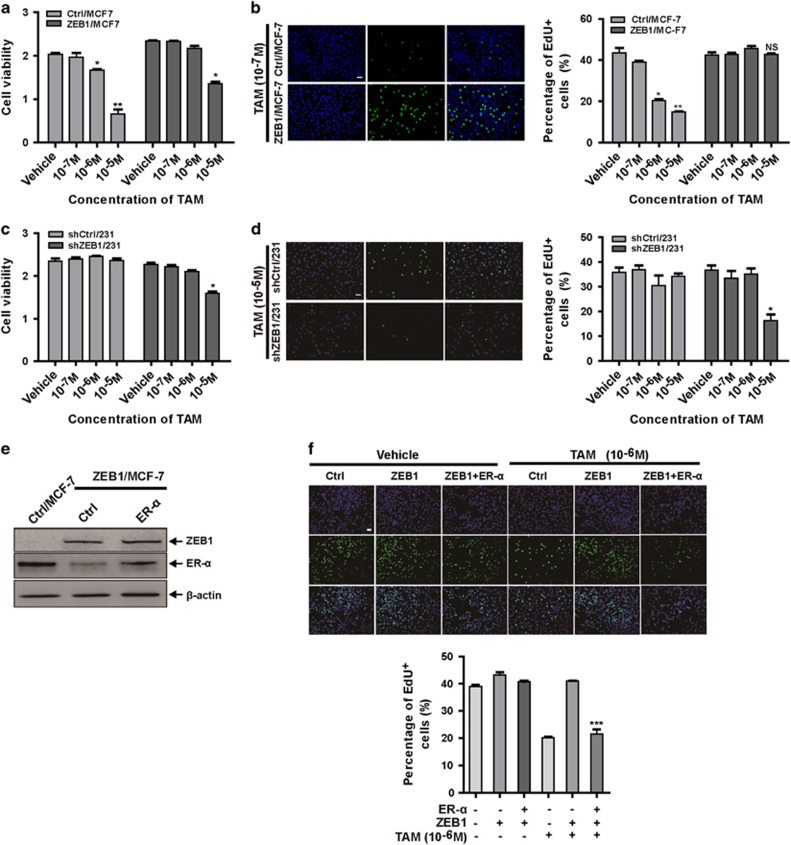
Ectopic ZEB1 confers antiestrogen resistance in breast cancer cells. (**a** and **b**) ZEB1/MCF-7 and Ctrl/MCF-7 cells were treated with different concentrations of tamoxifen for 72 h. Cell growth inhibition was determined by cell viability (**a**) and EdU proliferation (**b**) assays. *P<0.05, **P<0.01 *versus* the respective control in one-way analysis of variance (ANOVA) followed by Tukey's honestly significant difference test. (**c** and **d**) shZEB1/231 and shCtrl/231 cells were treated with different concentrations of tamoxifen for 72 h. Cell growth inhibition was determined by cell viability (**c**) and EdU proliferation (**d**) assays. *P<0.05 *versus* the respective control in one-way ANOVA followed by Tukey's honestly significant difference test. (**e** and **f**) An ER-*α* expression plasmid was transfected into ZEB1/MCF-7 cells, which were then treated with 10^−6^ M tamoxifen for 72 h. (**e**) The expression of ZEB1 and ER-*α* were assessed by immunoblotting and normalized to the levels of *β*-actin. (**f**) Cell growth inhibition was determined by EdU proliferation assays. ***P<0.001 *versus* the respective control in one-way ANOVA followed by Tukey's honestly significant difference test

To further determine whether ZEB1-induced loss of ER-*α* caused antiestrogen resistance in breast cancer cells, ZEB1/MCF-7 or shZEB1/231 cells were treated with tamoxifen, and cell viability was measured. ZEB1 overexpression rendered MCF-7 cells less sensitive to tamoxifen treatment (Figure 5a). An 5-ethynyl-2′-deoxyuridine (EdU) cell proliferation assay further revealed that the overexpression of ZEB1 markedly increased the number of cells in the S phase after treatment with tamoxifen (Figure 5b). The percentage of cells in S phase increased from 14.75% in Ctrl/MCF-7 to 43.42% in ZEB1/MCF-7 cells after the addition of 10^−6^ M tamoxifen. Conversely, ZEB1 depletion in MDA-MB-231 cells decreased cell viability (Figure 5c) and the percentage of cells in S phase (Figure 5d) upon treatment with tamoxifen. These results were not unique to tamoxifen; ZEB1 overexpression in MCF-7 cells and ZEB1 knockdown in MDA-MB-231 cells also reduced and enhanced, respectively, cell sensitivity to fulvestrant (Supplementary Figure S3), revealing that ectopic ZEB1 confers antiestrogen resistance in breast cancer cells.

In addition, we investigated whether ER-*α* downregulation is important for ZEB1-induced resistance to antiestrogen treatment. An ER-*α* expression plasmid was transfected into ZEB1/MCF-7 cells (Figure 5e) prior to treatment with tamoxifen. The EdU cell proliferation assay showed that the rescue of ER-*α* expression in ZEB1/MCF-7 restored sensitivity to tamoxifen (Figure 5f) and fulvestrant (Supplementary Figure S4), which demonstrated that the downregulation of ER-*α* is involved in ZEB1-mediated antiestrogen resistance.

### Downregulation of ZEB1 increases the antiestrogen sensitivity of breast cancer *in vivo*

**Figure 6:**
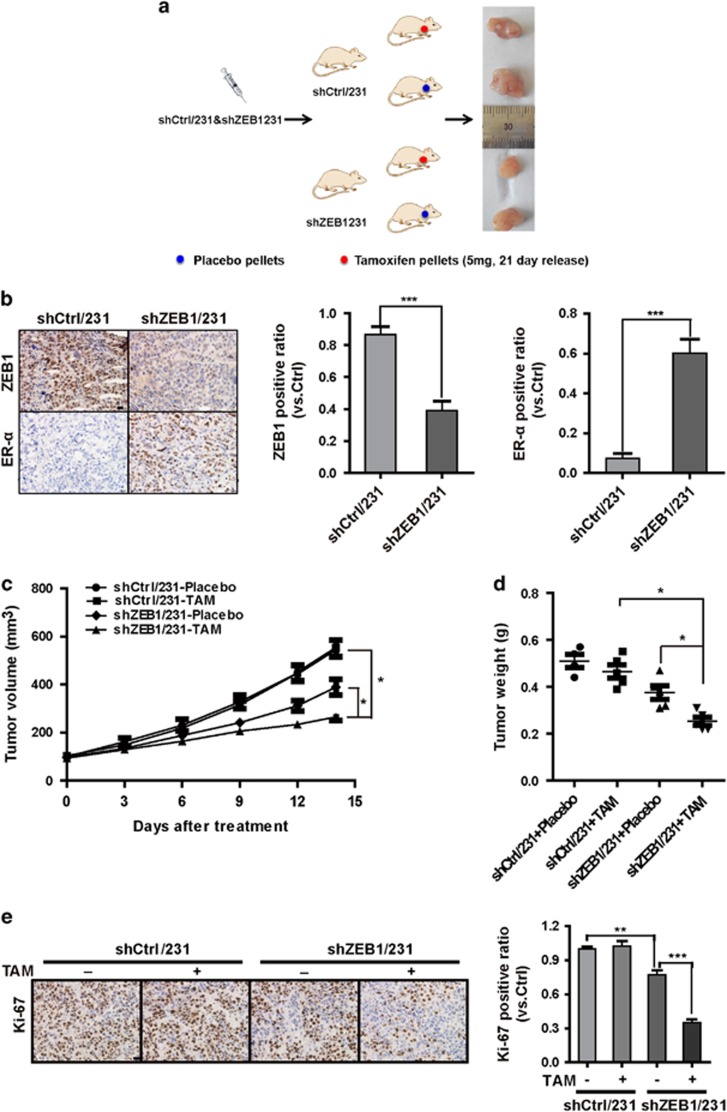
Downregulation of ZEB1 increases antiestrogen sensitivity *in vivo* in a nude mouse xenograft model. (**a**) A total of 1 × 10^6^ shZEB1/231 or shCtrl/231 cells were injected into the mammary fat pads of nude mice. When the tumor volume was ~100 mm^3^, the mice were divided into two groups (*n*=5) and treated with tamoxifen and placebo, respectively. (**b**) The expression of ZEB1 and ER-*α* in shZEB1/231 and shCtrl/231 xenograft tumors was examined by immunohistochemical staining. ***P<0.001 *versus* the respective control in Student's *t*-test. Scale bars, 20 *μ*m. (**c** and **d**) Approximate tumor volumes (**c**) and weights (**d**) were measured. *P<0.05 *versus* the respective control in Student's *t*-test. (**e**) The expression of Ki-67 in tamoxifen- or placebo-treated xenograft tumors was examined by immunohistochemical staining. **P<0.05, ***P<0.001 *versus* the respective control in Student's *t*-test. Scale bars, 20 *μ*m

Next, we assessed whether ZEB1 downregulation in breast cancer cells would influence tumor response to antiestrogen treatment *in vivo*. shZEB1/231 or shCtrl/231 cells were injected into the mammary fat pads of female BALB/c nude mice to establish tumors as a xenograft model, and mice were subsequently treated with tamoxifen (Figure 6a). Immunohistochemical staining confirmed the downregulation of ZEB1 and the upregulation of ER-*α* in tumors from shZEB1/231 mice compared to the shCtrl/231 mice (Figure 6b). Notably, upon treatment with tamoxifen, tumor volumes and weights were significantly decreased in mice injected with shZEB1/231 cells compared with those of mice injected with shCtrl/231 cells (Figures 6c and d). Ki-67 expression was also reduced in tumors from shZEB1/231 mice compared to that of shCtrl/231 mice (Figure 6e). Conversely, ZEB1 overexpression in MCF-7 cells resulted in decreased cell sensitivity to tamoxifen in tumor xenografts (Supplementary Figure S5). These data collectively suggest a major role for ZEB1 in affecting the responsiveness of breast cancer cells to antiestrogen treatment *in vivo*.

## Discussion

Resistance to antiestrogen therapy is one of the major barriers to the successful treatment of breast cancer, and ER-*α* expression is currently the main biomarker of response to antiestrogen treatment.^5, 6, 9, 40, 41^ It has been well established that a combination of genetic, epigenetic, and transcriptional controls regulates ER-*α* expression. However, the ontogeny of tumor progression that leads to the formation of the ER-*α*-negative and/or antiestrogen-resistant state is not clearly understood. Our work reveals a key role for ZEB1 in antiestrogen resistance in breast cancer. First, we found that ZEB1 interacts with DNMT3B and HDAC1 at the ER-*α* promoter, leading to DNA hypermethylation and the downregulation of ER-*α* in breast cancer cells. Second, ZEB1 expression is higher than and is inversely correlated with the amount of ER-*α* protein in breast cancer patients. Third, the downregulation of ZEB1 considerably increases the responsiveness of breast cancer cells to antiestrogen therapy *in vitro* and *in vivo*, and this effect was ER-*α* dependent. Therefore, our study indicates that ZEB1 may act as a determinant of antiestrogen resistance in breast cancer.

Although adjuvant antiestrogen therapy is recommended for all women with ER-*α*-positive breast cancer, >50% of ER-*α*-positive tumors that initially respond to antiestrogen treatment will eventually develop resistance.^8, 9, 10, 41^ Possible causes for the intrinsic and acquired resistance include the pharmacological properties of antiestrogen treatments, alterations in the expression and function of ER-*α*, interactions of tumors with the local microenvironment, and genetic alterations of tumor cells.^42, 43, 44^ In the present study, we found that the transcriptional regulation of ER-*α* can be modulated by ZEB1, a protein that is upregulated in high-grade breast cancer phenotypes.^27, 28^ Thus, the abundance of ER-*α* protein is relatively low in tumors with high ZEB1 expression. These cases, despite their ER-*α*-positive status, are less likely to benefit from antiestrogen treatment. The importance of this finding is that the loss of ER-*α* in breast cancer patients is indicative of a poor prognosis,^10, 13^ and this reduced expression or absence of ER-*α* caused by ZEB1 may have implications for ER-*α*-negative and/or antiestrogen-resistant breast cancers. Our findings help to explain why some ER-*α*-positive tumors respond poorly to antiestrogen treatment. Therefore, restoring ER-*α* expression by inhibiting ZEB1 provides a potential therapeutic strategy for restoring antiestrogen sensitivity in breast cancer.

A number of causes have been identified to account for ER-*α* inactivation, such as homozygous deletion, loss of heterozygosity, or *ER* gene mutation.^14, 15, 16, 17, 18, 19^ Here, we further demonstrated that ectopic ZEB1 represses ER-*α* expression in breast cancer cells and thus recapitulates a loss of response to antiestrogen treatment *in vitro* and *in vivo*. Mechanistically, ZEB1 interacts with DNMT3B and HDAC1 at the ER-*α* promoter, causing promoter hypermethylation, histone deacetylation, and transcriptionally silenced chromatin.^20, 21, 22^ We were able to reverse this state using the methylation inhibitor AZA and the HDAC inhibitor VPA. The binding of ZEB1 to the ER-*α* promoter in breast cancer cells provided further evidence that the downregulation of ER-*α* by ZEB1 is a possible functional mechanism for eliminating ER-*α* in ZEB1-positive breast cancer cells. Therefore, these results indicate that ZEB1 can regulate ER-*α* expression by inducing promoter methylation and chromatin remodeling to achieve transcriptional repression.^35, 36, 37, 45^

Functional hallmarks of ER-*α*-negative cells include their ability to proliferate without estrogen stimulation and their antiestrogen resistance.^10, 18^ This state is consistent with our results in the present study showing that ZEB1/MCF-7 cells were resistant to tamoxifen and fulvestrant without significant G_1_/S arrest compared to Ctrl/MCF-7 cells. Moreover, ZEB1 tumors were able to grow without estrogen (data not shown) and were unaffected by tamoxifen treatment in the xenografted nude mice. These experiments strongly indicated that ZEB1 expression caused estrogen independence in breast cancer cells that possibly led to antiestrogen resistance. Notably, ZEB1 expression is increased in high-grade human breast tumors, and a high percentage of these tumors are ER-*α* negative.^27^ We observed similar results in breast cancer patients; a negative correlation exists between ZEB1 and ER-*α* expression. Therefore, ZEB1 overexpression may provide a mechanistic link between the development of aggressive breast cancer and the loss of ER-*α* expression and may provide a method to elucidate the ontogeny of ER-*α*-negative and/or antiestrogen-resistant breast cancer.

In this study, we demonstrated an alternative mechanism for ZEB1/ER-*α*–mediated antiestrogen resistance in breast cancer that supplements the promoter methylation and deacetylation of ER-*α*. Considering that estrogen induces ZEB1 expression while regulating breast cancer development and progression,^45^ we therefore suggest a potential role for ZEB1 at an intersection between intrinsic and acquired resistance to antiestrogen therapy. The inhibition of ZEB1 to restore ER-*α* expression, in combination with methylation inhibitors and/or HDAC inhibitors, will represent a new strategy for overcoming antiestrogen resistance in breast cancer.

## Materials and Methods

### Cell culture and transfection

Human breast cancer cell lines were maintained in DMEM (MDA-MB-231, MCF-7, and SUM-159) and RPMI 1640 (ZR-75-1) supplemented with 10% fetal bovine serum, 100 IU penicillin, and 100 mg/ml streptomycin. For antiestrogen treatment, cells were cultured in phenol red-free minimal essential media with 5% fetal bovine serum and then treated with different concentrations of tamoxifen or fulvestrant. Cells were transfected using Lipofectamine 2000 (Invitrogen, CA, USA) according to the manufacturer's protocol.

### Plasmid construction

The human complementary DNA fragment encoding full-length ZEB1^37^ was prepared by PCR and cloned into pLV-EF1-MCS-IRES-Bsd (Biosettia, San Diego, USA). The lentiviral-based vector pLV-H1-EF1*α*-puro (Biosettia, San Diego, USA) was used to express shRNAs in breast cancer cells. The human ER-*α* promoter (-4184/-1951) sequence was obtained by PCR from human genomic DNA and cloned into the pGL3-promoter vector (Promega, Wisconsin, USA). Mutagenesis of E_2_-boxes I and II in the human ER-*α* promoter was performed using a QuikChange Lightning Site-Directed Mutagenesis kit (Stratagene, Santa Clara, USA). Primer sequences are listed in Supplementary Data.

### Generation of lentiviruses

Lentiviruses were generated by transfecting subconfluent HEK293T cells with lentiviral vectors and packaging plasmids by calcium phosphate transfection. Viral supernatants were collected 48 h after transfection, centrifuged at 75 000 × *g* for 90 min, resuspended and filtered through 0.45-*μ*m filters (Millipore, MA, USA).

### Methylation assays

DNA was extracted from ZEB1-expressing or ZEB1-silenced breast cancer cells and processed for bisulfite treatment (Qiagen, Hilden, Germany). Bisulfite-treated DNA was then used to examine the methylation status of the CpG islands in the ER-*α* promoter using bisulfite sequencing PCR and methylation-specific PCR according to the manufacturer's protocol (Roche, Basel, Switzerland). Primer sequences are listed in Supplementary Data.

### RNA extraction and quantitative RT-PCR

Cells were transfected with the ZEB1 expression plasmid or ZEB1-targeted shRNA. Total RNA (0.5 *μ*g) from each sample was collected using TRIzol reagent (Invitrogen), and for first-strand complementary DNA synthesis was performed using M-MLV Reverse Transcriptase (Takara, Tokyo, Japan). The specific products of ZEB1 and ER-α were amplified by quantitative PCR using a TransStart Green Q-PCR SuperMix kit (TransGen, Beijing, China). GAPDH was used as a normalization control. Primer sequences are listed in Supplementary Data.

### Immunoblotting assay

The preparation of total cell extracts and immunoblotting with appropriate antibodies were performed as previously described.^37^ The appropriate antibodies were used as indicated in Supplementary Data. Labeled proteins were visualized with an ECL chemiluminescence kit (Millipore).

### Luciferase assay

Cells were co-transfected with the wild-type or mutant human ER-*α* promoters and ZEB1 expression plasmid in 24-well plates. Lysates were prepared 36 h after transfection, and luciferase activity was measured using the Dual-Luciferase Reporter Assay System (Promega, Wisconsin, USA) according to the manufacturer's protocols. Luciferase activity was normalized to the values for Renilla luciferase.

### Immunoprecipitation assay

Cell lysates were incubated with specific antibodies and Protein G agarose beads (Invitrogen) at 4 °C overnight, followed by three washes with a buffer containing 50 mM Tris (pH 7.5), 100 mM NaCl, 7.5 mM EGTA, and 0.1% Triton X-100. The antibodies used for immunoprecipitation are shown in Supplementary Data.

### Chromatin immunoprecipitation

ChIP assays were performed using an EZ-ChIP kit (Millipore) according to the manufacturer's instructions. The antibodies used in these experiments are shown in Supplementary Data. The fragments of human ER-*α* promoter containing the E_2_-box I and II elements in immunoprecipitates were amplified by quantitative PCR.

### Tissue samples

A total of 248 breast cancer subjects were obtained from the General Hospital of the People's Liberation Army (PLAGH, Beijing, China). All patients had histologically confirmed invasive ductal carcinoma of the breast and were recruited by the same department. This study was approved by the institutional ethics committees at PLAGH and the Medical College of Nankai University.

### Immunohistochemical analysis

Immunohistochemical analysis of paraffin-embedded sections was performed using the Envision Kit (Dako, Denmark) according to the manufacturer's protocol. Sections were boiled in retrieval solutions to expose antigens. The specific antibodies (see Supplementary Data) were applied to the sections. Slides were counterstained with hematoxylin, dehydrated, and mounted. Immunostaining was independently evaluated by 2 pathologists.

### Cell proliferation assay

Cells were seeded onto a 96-well plate at a density of 4 × 10^3^ cells/well and treated with different concentrations of tamoxifen or fulvestrant for 24–96 h. Cell viability was assessed using the CCK-8 assay according to the manufacturer's protocols (Dojindo, Tokyo, Japan). Six parallel replicates were measured for each sample.

### 5-ethynyl-2′-deoxyuridine cell proliferation assay

Cells growing in 24-well plates were cultured in the presence of tamoxifen or fulvestrant at the indicated time points and then assayed with the Cell-Light EdU Apollo488 *In Vitro* Imaging Kit according to the manufacturer's instructions (RiboBio, Guangzhou, China). Images were taken and analyzed using a Confocal FV1000 microscope (Olympus, Tokyo, Japan). EdU-positive cells were calculated as (EdU add-in cells/Hoechst stained cells) × 100%. At least 200 cells were counted per well.

### Tumor xenograft experiments

All experimental procedures involving animals were performed according to the institutional ethical guidelines for animal experiments and approved by the Ethics Committee for Animal Use at the Medical College of Nankai University. In brief, cells were collected and suspended in 200 *μ*l phosphate-buffered saline at a concentration of 5 × 10^6^ cells/ml and then injected into the mammary fat pads of female BALB/c nude mice. Tumor development was allowed to reach a volume of ~100 mm^3^. The mice were then randomized into 2 groups (5 mice per group), and tamoxifen pellets (5 mg/pellet, Innovative Research of America) or placebo pellets were subcutaneously embedded for another 3 weeks. Tumor volumes (*V*) were calculated by measuring the length (*L*) and width (*W*) of the tumor with calipers and applying the following formula: *V*=(*L* × *W*^*2*^) × 0.5. Tumor tissues were also processed and sectioned for histological evaluation.

### Statistical analysis

Statistical analyses were performed using SPSS 13.0 software (SPSS, Chicago, IL, USA). Data are presented as the means±S.D. and represent three independent experiments. Spearman's rank correlation test was used to analyze the correlation of gene expression in tissue samples. One-way analysis of variance was used to compare means between treatment groups. Where appropriate, Student's *t*-test for unpaired observations was applied. A *P*-value<0.05 was considered significant.
