# Supplementary material for: ZEB1 induces ER-α promoter hypermethylation and confers antiestrogen resistance in breast cancer
Source: Cell Death Dis. 2017 Apr 6;8(4):e2732–. doi: 10.1038/cddis.2017.154 (PMC5477580; doi:10.1038/cddis.2017.154)
Supplement: Supplementary Information [file cddis2017154x1.pdf]

Supplementary materials and methods

Primers

Construction of full-length ZEB1 expression vector	
forward	5'-ATGGCGGATGGCCGCC-3'
reverse	5'-TTAGGCTTCATTTGTCTTTTCTTCA-3'
Construction of shRNA vector	
shZEB1-1	5'-CGGCGCAATAACGTTACAAAT-3'
shZEB1-2	5'-GGCGCAATAACGTTACAAA-3'
shZEB1-3	5'-CCTCTCTGAAAGAACACATTA-3'
shDNMT3B-1	5'-GCCTCAAGACAAATTGCTA-3'
shDNMT3B-2	5'-GCTGTCCGAACTCGAAATAAC-3'
shDNMT3B-3	5'-GGTTTGGCGATGGCAAGTTCT-3'
shHDAC1-1	5'-GGAAAGTCTGTTACTACTA-3'
shHDAC1-2	5'-GCTCCATCCGTCCAGATAACA-3'
shHDAC1-3	5'-GCTGGCAAAGGCAAGTATTAT-3'
shER-α-1	5'-GGAGAATGTTGAAACACAAGC-3'
shER-α-2	5'-GGATTTGACCCTCCATGATCA-3'
shER-α-3	5'-GCATTCTACAGGCCAAATT-3'
Construction of ER-α promoter	
ER-wtE_2 forward	5'-CGGGGTACCATTATGGCTCCGTTGTCG-3'
ER-wtE_2 reverse	5'-CCGCTCGAGATGGAGGGCTGGCTGTAC-3'
Site-Directed Mutagenesis of ER-α promoter	
ER-mE_2I forward	5'-TGCTATAAAAACCAGCA**TC**TGGGGCTGGACGCAG-3'
ER-mE_2I reverse	5'-CTGCGTCCAGCCCCAGATGCTGGTTTTTATAGCA-3'
ER-mE_2II forward	5'-TGGCGGCGGGCA**TC**TGTAGTCCCAG-3'
ER-mE_2II reverse	5'-CTGGGACTACAGATGCCCGCCGCCA-3'
Bisulfite sequencing PCR	
BSP forward	TATTGAAAAGTGGATTATTGATGGAT
BSP reverse	ATCACCCAAACTAAAATACAATAAC
Methylation specific PCR	
m-MSP forward	AAAAATTAGTATTTGGGGTTGGAC
m-MSP reverse	CCATATTAACCGAAATAATCTCGAT
u-MSP forward	AAATTAGTATTTGGGGTTGGATGT
u-MSP reverse	TTCACCATATTAACCAAAATAATCTCA
Quantitative RT-PCR	
human ZEB1 forward	5'-CAGCTTGATACCTGTGAATGGG-3'
human ZEB1 reverse	5'-TATCTGTGGTCGTGTGGGACT-3'
human ER-α forward	5'-ATGTGGCTAGTTTGTCCTC-3'
human ER-α reverse	5'-AGCAAGATTTCCTCCAGGTC-3'
Quantitative ChIP	
E_2-box I forward	5'-TATGGCTCCGTTGTCG-3'

E_2-box I reverse	5’-GGGATTACAGGCGTGAG-3’
E_2-box II forward	5’-GGCACCTGTAGTCCCA-3’
E_2-box II reverse	5’-CCATCTCGGCTCACTG-3’
GAPDH forward	5’-GGAGCGAGATCCCTCCAAAAT-3’
GAPDH reverse	5’-GGCTGTTGTCATACTTCTCATGG-3’
Gene-free forward	5’-ATGGTTATGTAGCCTTTGG-3’
Gene-free reverse	5’-GAGGAATACGGTATGGAA-3’

Antibodies

Marker	Species	Application	Manufacturer	Catalog No.	Dilution
anti-ZEB1	Rabbit	IHC	Abcam	ab87280	1:100
	Rabbit	IB	Santa Cruz	sc-25388	1:1000
	Rabbit	IP	Proteintech	221544-1-AP	2 μg/reaction
		CHIP			1 μg/reaction
anti-ER-α	Rabbit	IHC	CST	#8644	1:100
		IB			1:1000
anti-PR	Rabbit	IHC	Abcam	ab16661	1:100
anti-Ki67	Rabbit	IHC	Abcam	ab131492	1:1000
anti-DNMT3B	Mouse	IP	Abcam	ab13604	2 μg/reaction
		CHIP			1 μg/reaction
		IB			1:1000
anti-HDAC1	Rabbit	IP	Abcam	ab51846	2 μg/reaction
		CHIP			1 μg/reaction
		IB			1:1000
anti-β-actin	Mouse	IB	Sigma	A-4700	1:5000

IHC: Immunohistochemistry; IB: Immunoblotting; IF: Immunofluorescence; IP: Immunoprecipitation; ChIP: Chromatin immunoprecipitation

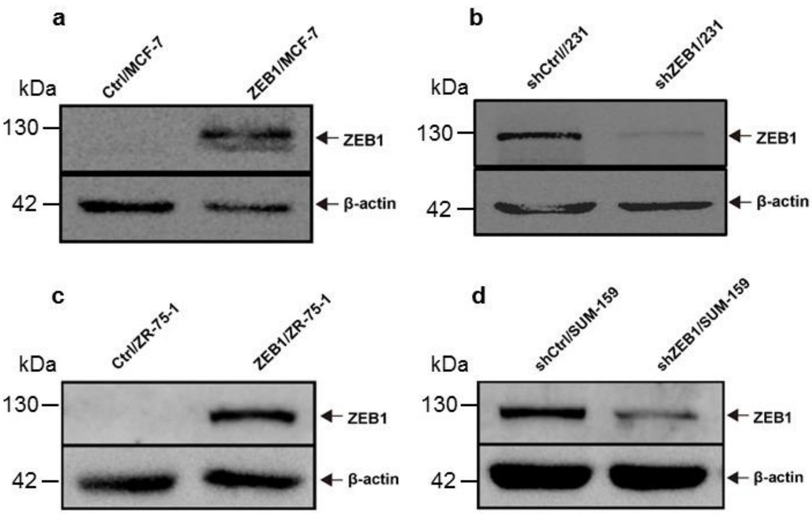

Figure S1 Gain- or loss-of-function of ZEB1 in breast cancer cells. (**a,c**) MCF-7 (**a**) and ZR-75-1 (**c**) cells were stably transfected with the ZEB1 expression plasmid. The expression of ZEB1 was verified by immunoblotting and normalized to the levels of β-actin. (**b,d**) MDA-MB-231 (**b**) and SUM-159 (**d**) cells were stably transfected with the specific shRNA targeting ZEB1. The expression of ZEB1 was verified by immunoblotting and normalized to the levels of β-actin.

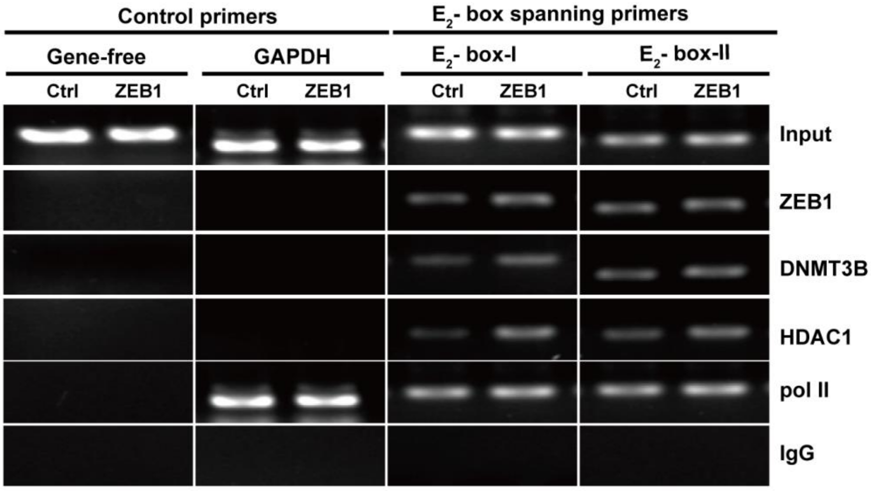

Figure S2 ZEB1 recruits to the ER-α promoter in an E_2-box dependent manner by forming a complex with HDCA1 and DNMT3B. The association of ZEB1, HDAC1, DNMT3B, and RNA polymerase II (Pol II) with the ER-α promoter was analyzed by ChIP assay. The amplified sequence of the ER-α promoter fragment containing E2-box I and E2-box II elements is shown. GAPDH and a "gene-free" fragment was used as positive and negative controls, respectively. Input DNA amounts were confirmed by equal loading of chromatin.

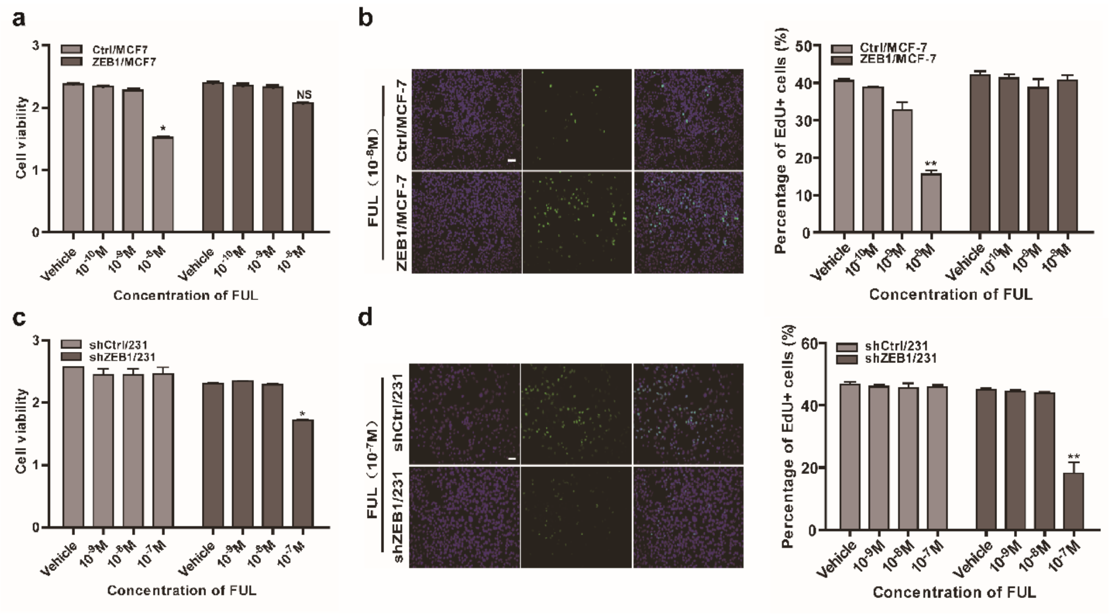

Figure S3 Ectopic ZEB1 confers antiestrogen resistance in breast cancer cells. (**a** and **b**) ZEB1/MCF-7 and Ctrl/MCF-7 cells were treated with different concentrations of fulvestrant for 72 h. Cell growth inhibition was determined by cell viability (**a**) and EdU proliferation (**b**) assays. *$P < 0.05$, **$P < 0.01$ vs. the respective control in one-way ANOVA followed by Tukey's honestly significant difference test. (**c** and **d**) shZEB1/231 and shCtrl/231 cells were treated with different concentrations of fulvestrant for 72 h. Cell growth inhibition was determined by cell viability (**c**) and EdU proliferation (**d**) assays. *$P < 0.05$, **$P < 0.01$ vs. the respective control in one-way ANOVA followed by Tukey's honestly significant difference test.

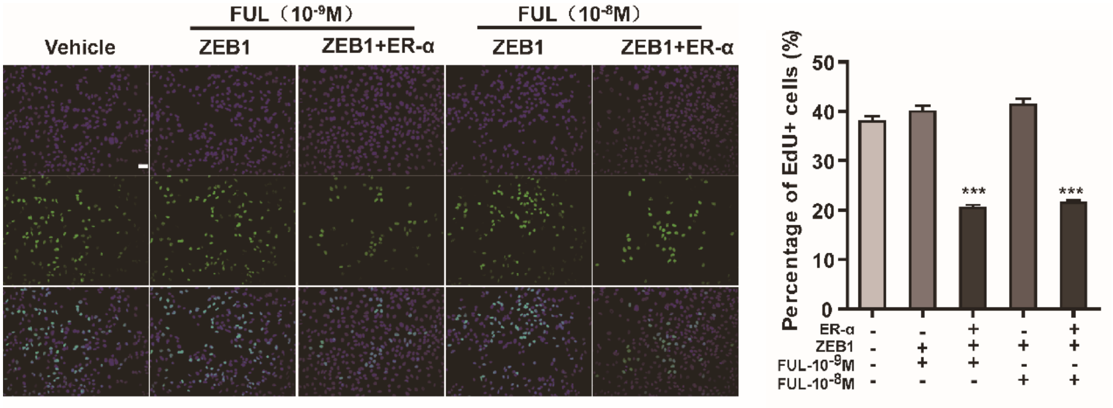

Figure S4 ZEB1 induces antiestrogen resistance in an ER-α-dependent manner. An ER-α expression plasmid was transfected into ZEB1/MCF-7 cells, which were then treated with 10^{-8} M and 10^{-9} M fulvestrant for 72 h, respectively. Cell growth inhibition was determined by EdU proliferation assays. ****P* < 0.001 vs. the respective control in one-way ANOVA followed by Tukey's honestly significant difference test.

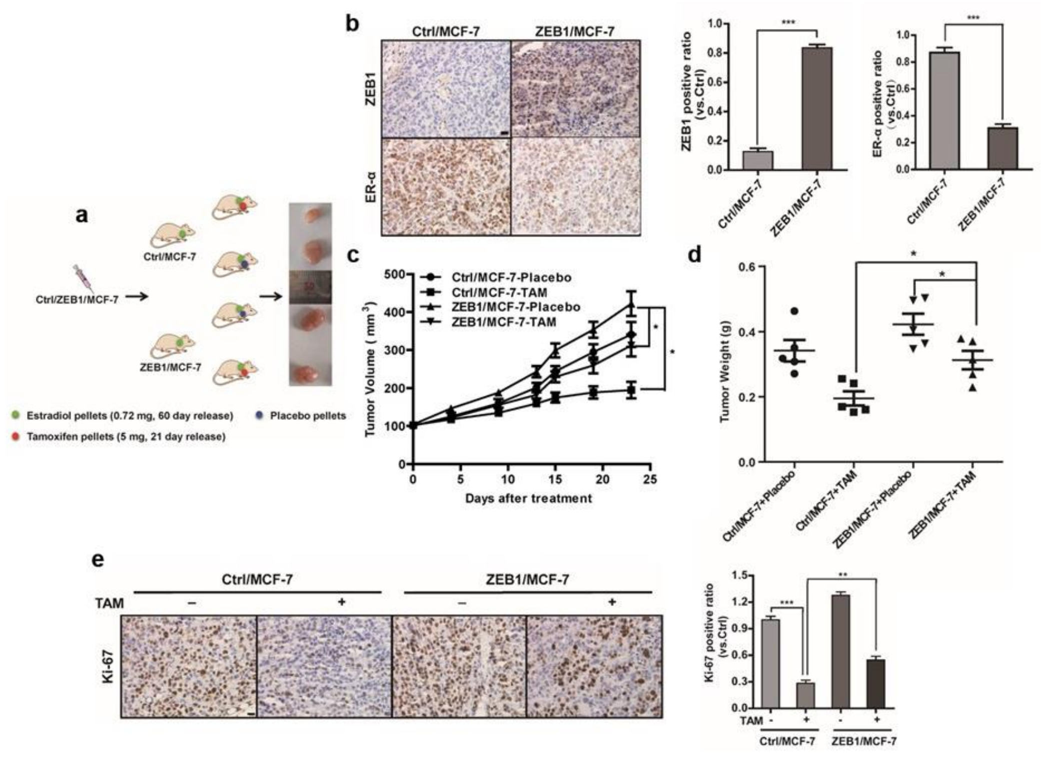

Figure S5 Ectopic ZEB1 decreases antiestrogen sensitivity *in vivo* in a nude mouse xenograft model. (**a**) A total of 1.5×10^6 Ctrl/MCF-7 or ZEB1/MCF-7 cells were injected into the mammary fat pads of nude mice. The tumors were allowed to grow 10 days in the presence of estradiol. The mice were then divided into two groups (n=5) and treated with tamoxifen and placebo, respectively. (**b**) The expression of ZEB1 and ER-α in Ctrl/MCF-7 or ZEB1/MCF-7 xenograft tumors was examined by immunohistochemical staining. ***$P < 0.001$ vs. the respective control in Student's *t*-test. Scale bars, 20 μm. (**c** and **d**) Approximate tumor volumes (**c**) and weights (**d**) were measured. *$P < 0.05$ vs. the respective control in Student's *t*-test. (**e**) The expression of Ki-67 in tamoxifen- or placebo-treated xenograft tumors was examined by immunohistochemical staining. **$P < 0.01$, ***$P < 0.001$ vs. the respective control in Student's *t*-test. Scale bars, 20 μm.
